# Multidimensional schizotypy and embodied emotions

**DOI:** 10.3389/fpsyg.2023.1141799

**Published:** 2023-04-25

**Authors:** Lénie J. Torregrossa, Scott D. Blain, Matthew A. Snodgress, Sohee Park

**Affiliations:** ^1^Department of Psychology, Vanderbilt University, Nashville, TN, United States; ^2^Department of Psychiatry, Vanderbilt University Medical Center, Nashville, TN, United States; ^3^Department of Psychiatry, University of Michigan, Ann Arbor, MI, United States; ^4^Department of Psychology, University of Minnesota, Minneapolis, MN, United States

**Keywords:** bodily self, schizotypy, emotion awareness, embodiment, body maps, self-disturbances (Ichstörungen)

## Abstract

**Background:**

Disembodiment and socio-emotional deficits are core features of the schizophrenia spectrum from the prodromal stages to chronic illness. A recent study documented anomalous emotional embodiment in individuals with schizophrenia. Although bodily self disturbances have been shown to precede and predict psychosis onset in at-risk populations, the etiology of anomalous emotional embodiment remains largely unexplored. The present study investigated bodily maps of emotions in relation to schizotypy to extend our understanding of embodied emotions in the schizophrenia spectrum.

**Methods:**

A total of 419 participants (312 female; 19.50 ± 1.22 years) completed a topographical body mapping task where they reported patterns of embodiment experienced in the context of eleven different emotions and a neutral state (EmBODY). Embodied emotions were investigated in relation to multidimensional schizotypy.

**Results:**

Individuals with elevated negative schizotypy experienced embodied emotions with higher intensity (*r* = 0.16, *p* = 0.003) but lower clarity (i.e., endorsing activation and deactivation in the same bodily location; β = −0.28, 95% CI [−0.54, −0.03], *Z* = 2.25, *p*=0.02) and endorsed more incongruent bodily sensations of emotions (i.e., reporting bodily activation in the context of a low-arousal emotion, *r* = 0.12, *p* = 0.05; reporting bodily deactivation in the context of high-arousal emotions, *r* = 0.13, *p* = 0.02). In line with the anomalous emotional embodiment documented in individuals with schizophrenia, some of these differences were particularly notable for low-arousal emotions.

**Discussion:**

These results reveal negative schizotypy as a significant correlate of differences in emotional embodiment. More work is needed to link these differences to the anomalous bodily sensations of emotions documented in schizophrenia and assess their functional impact.

## 1. Introduction

The body is the physical anchor for social and emotional experiences. In fact, the subjective experience of our emotions depends on our perception of physiological changes occurring in the body (James, 1884; Ekman, 1992; Damasio and Carvalho, 2013). Recognition and expression of emotional states is, in turn, crucial to communicating internal sensations and intentions to others (Ekman and Oster, 1979; Keltner et al., 2019). Using a computerized mapping tool, Nummenmaa et al. (2014) showed that different emotional experiences are associated with unique patterns of bodily sensations (e.g., widespread peripheral deactivation in the context of depression, activation of the face, upper chest, and arms and hands in the context of anger), and statistical analyses confirmed the independence of the topographical representation of each of the 14 emotions explored. A recent study using the same tool found evidence for the universality of these bodily sensation maps of emotions by showing concordant bodily maps of emotions across different cultures (Volynets et al., 2020). Thus, distinct “bodily signatures” of emotions, analogous to the unique neural signatures of basic affective states (Saarimäki et al., 2016), were identified in the general population.

Anomalous embodiment and socio-emotional deficits have long been considered central to schizophrenia. Kraepelin and Bleuler believed that “flat” and “inappropriate” affect were core features of the illness (see Trémeau, 2006 for a review). Consistent with these early theoretical models, empirical evidence showed that the facial expression of emotions is reduced in individuals with schizophrenia (Berenbaum and Oltmanns, 1992; Kring and Moran, 2008) though the recruitment of facial muscles underlying the production of facial expressions was found to be intact (Kring et al., 1999). The shared circuit hypothesis posits that brain regions recruited during the processing of one’s own emotional experiences are also involved in the processing of the emotional experiences of others (Singer, 2006; Tschacher et al., 2017). Thus, our ability to infer the emotional states of others stems from our capacity to internally simulate and re-create the bodily states of others, which highlights the role of emotional embodiment in understanding other’s affective states. Research on automatic facial mimicry in schizophrenia has shown mixed results, with early investigations indicating impaired facial mimicry (Berndl et al., 1986; Steimer-Krause et al., 1990; Varcin et al., 2010; Sestito et al., 2013), and more recent studies documenting intact automatic facial mimicry in this population (Chechko et al., 2016; Raffard et al., 2018; Riehle and Lincoln, 2018; Torregrossa et al., 2019a). Despite the inconsistency of these results, a recent review concluded that individuals with schizophrenia exhibit reduced and more variable facial mimicry (e.g., unusual pattern of corrugator and zygomaticus activity in response to emotional stimuli; Dean et al., 2021).

At the whole-body level, a recent study found evidence for anomalous emotional embodiment in individuals with schizophrenia (Torregrossa et al., 2019b). Specifically, individuals with schizophrenia reported less differentiated and less congruent bodily sensations of emotions (e.g., experience of bodily deactivation in the context of high-arousal emotions). This study also implemented a similarity analysis, which indicated a specific alteration of embodiment for low-arousal emotions in schizophrenia. Findings also suggested that the type of bodily sensations of emotions (i.e., activation or deactivation) was less clearly defined in schizophrenia such that individuals with schizophrenia reported more “mixed” (i.e., simultaneous experiencing activation and deactivation in the same bodily region) bodily sensations of emotions, though there was no group difference in the intensity of reported embodied emotions (Torregrossa et al., 2019b). These differences in emotional embodiment in schizophrenia were recently confirmed across different cultures (Lee et al., 2022). Additionally, anomalous emotional embodiment has been linked to interoceptive deficits in schizophrenia (Ardizzi et al., 2016, Koreki et al., 2021; Torregrossa et al., 2022; Yao and Thakkar, 2022).

Though bodily self disturbances have been shown to precede and predict psychosis onset in at-risk populations (Nelson et al., 2008, 2012), the etiology of anomalous emotional embodiment remains largely unexplored. While one recent study documented decreased bodily activation and increased bodily deactivation in the context of emotions in individuals with high hallucination proneness (Thomas et al., 2021), the link between emotional embodiment and other aspects of psychosis-proneness remains unknown. Schizotypy refers to personality traits and experiences related to subthreshold psychotic experiences (Debbané et al., 2015) that imply a latent liability for schizophrenia (Lenzenweger, 2018). Schizotypy is multidimensional, consisting of three major syndromes, analogous to the positive, negative, and disorganized symptoms of schizophrenia (Raine et al., 1994). An advantage to studying embodiment and bodily self-disturbances in relation to schizotypy is the ability to circumvent the potential confounds of medication effects in those diagnosed with schizophrenia. Interestingly, contemporary research exploring different aspects of the bodily self in relation to schizotypy have shown varied results. For instance, while no link was found between interoceptive functioning and schizotypy (Torregrossa et al., 2022), schizotypy was linked to disruptions in body ownership (Thakkar et al., 2011; Germine et al., 2013; Torregrossa and Park, 2022) and agency deficits (Asai and Tanno, 2007, 2008; Moore et al., 2020). To our knowledge, emotional embodiment has never been studied in relation to schizotypy.

The present study used a computerized body mapping task to explore emotional embodiment in relation to the three schizotypy dimensions in an effort to understand the role of embodiment in psychosis-proneness. We hypothesized that the bodily maps of emotions generated by our sample would be consistent with those of previous studies assessing embodied emotions in the general population (e.g., Nummenmaa et al., 2014; Volynets et al., 2020). Based on the results of Torregrossa et al. (2019b) in patients with schizophrenia, we hypothesized that the diffusion (i.e., spread), size, and clarity (i.e., distinct areas of bodily activation vs. deactivation) of embodied emotions would be associated with schizotypy, especially for low-arousal emotions. We also expected schizotypy to predict incongruent bodily sensation of emotions (e.g., bodily activation for low arousal emotions). Given the lack of previous research in this area, no hypotheses concerning specific dimensions of schizotypy (i.e., positive, negative, and disorganized) were generated.

## 2. Materials and methods

### 2.1. Participants

A total of 419 participants (312 female; 19.50 ± 1.22 years) were recruited through the Vanderbilt University Psychology Subject Pool. Exclusion criteria were substance use or alcohol abuse within the past 6 months, brain injury, or neurological disease as we were interested in investigating embodiment of emotions in the neurotypical population without the potential confounds of neuropsychiatric disorders and psychotropic substance. All participants were English speakers and had normal or corrected-to-normal (i.e., 20/20) vision. Participants gave written informed consent, as approved by the Vanderbilt Institutional Review Board (#061100), and were granted course credit in compensation for their participation. Participants were recruited between 2016 and 2018. All participants completed the study in-person, in an experimental room where they completed the EmBODY task on a desktop computer (see details below) and self-report measures on paper. Demographic information is presented in Table 1.

**TABLE 1:** Demographic information for study participants.

	Participants (*N* = 419)
Gender (M/F/other)	103/312/1
Age	19.50 (1.22)
Handedness (R/L)	377/40
**Race/ethnicity**	
Black	35
Asian	91
Hispanic	13
Multiracial	41
White	227
Other	12

*Post hoc* power analyses revealed that, given our sample size of 419 participants, we had 80% power to detect a correlation of *r* = 0.137 and 90% power to detect a correlation of *r* = 0.158. Given that the average effect size reported in individual differences research is around *r* = 0.20 (Richard et al., 2003; Gignac and Szodorai, 2016) the current sample would be considered well powered for detecting our effects of interest.

### 2.2. Measures

#### 2.2.1. Emotional embodiment

##### 2.2.1.1. EmBODY task

**FIGURE 1:**
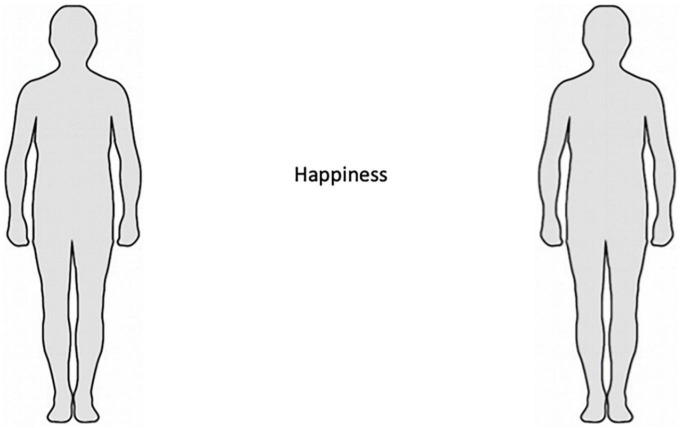
EmBODY task. Participants use the computer mouse to color where on their body they feel an increase **(left)** or decrease **(right)** in activity while experiencing a given emotion (e.g., happiness).

The EmBODY task (Nummenmaa et al., 2014) was used to assess embodied emotions. EmBODY (Figure 1) is a computerized body mapping tool requiring participants to color the bodily regions where they feel a change in activity while experiencing a given emotion. Specifically, EmBODY presents participants with two body outlines and an emotion word, and instructs them to color the bodily regions they feel activating (left body outline) and deactivating (right body outline) in the context of that emotion. Five basic emotions (anger, fear, disgust, happiness, and sadness), six non-basic emotions (anxiety, love, depression, pride, shame, and jealousy), and one neutral state were used in this study. The words were presented one at a time in a randomized order, and the participants were provided with a list specifying the definition of each emotion word.

In the EmBODY task, painting was dynamic such that repeated or extended painting over the same region increased the intensity of the coloring. Paint intensity at each pixel thus ranged from 0 (no paint) to 100 (fully saturated), representing the strength of the colored bodily sensation. The diameter of the painting tool was 12 pixels. Preprocessing consisted of manually screening for anomalous responding (i.e., writings, drawings, and absence of any painting) and smoothing the data using a Gaussian disk to remove spatial dependencies created by the mouse clicks during painting.

##### 2.2.1.2. Quantification of the characteristics of embodied emotions: diffusion, size, intensity, clarity, and congruency

In order to address our specific research questions regarding the characteristics (i.e., diffusion, size, intensity, clarity, and congruency) of emotional embodiment across schizotypy dimensions, we extracted several metrics from the EmBODY data. These methods were adapted from previous body mapping studies (Nummenmaa et al., 2014; Sachs et al., 2019; Torregrossa et al., 2019b; Galvez-Pol et al., 2021; Lloyd et al., 2021). In addition, we implemented a new metric to quantify the spatial spread of embodied sensations that we termed the *diffusion metric*.

To quantify the *diffusion* (i.e., spread) of embodied emotions, an individual’s body map for a given emotion was divided into five ROIs (i.e., arms, legs, head, abdomen, and chest) and diffusion scores were calculated for each. A diffusion score represents the average Manhattan distance (i.e., sum of the distances along each axis; Craw, 2011) between two colored pixels within an ROI. For instance, the Manhattan distance between colored pixel 1 at (*x*_1_, *y*_1_) and colored pixel 2 at (*x*_2_, *y*_2_) would be | *x*_1_ − *x*_2_| + | *y*_1_ − *y*_2_|. As such, a high diffusion score indicates a spread-out embodied emotion, while a low diffusion score represents a precisely defined one. Raw diffusion scores were converted to *Z*-scores.

To assess the *size* of bodily sensations, the proportion of the body colored for each emotion was computed (Galvez-Pol et al., 2021; Lloyd et al., 2021). Specifically, the number of non-zero pixels (i.e., colored pixels) was divided by the total number of pixels (50,364) for each participant, for each emotion. We note that this measurement does not take into consideration the intensity value of a pixel, but simply whether or not it was colored (i.e., 1 or 0). The proportion of body colored is conceptually close, though not analogous to, the diffusion metric previously described. In fact, by considering ROIs separately, the diffusion score represents the spread of a given bodily sensation. As such, a low diffusion score represents a precisely located bodily sensation (e.g., activation in the heart) while a low proportion of body colored represents a small bodily sensation which could be highly localized (e.g., activation in the heart only) or not (e.g., several dots of activation/deactivation across the body).

The *intensity* of the reported bodily sensations of emotions was estimated by summing the intensity values (ranging from 0 to 100 depending on click duration) of colored pixels per participant, per emotion. For the overall analysis, we averaged across emotions such that each participant had one intensity score estimating the overall strength of their emotional embodiment.

To investigate the *clarity* of embodied emotions, we computed the number of “mixed pixels” (i.e., pixels colored for both activation and deactivation by a given participant for the same emotion; Sachs et al., 2019; Torregrossa et al., 2019b).

**FIGURE 2:**
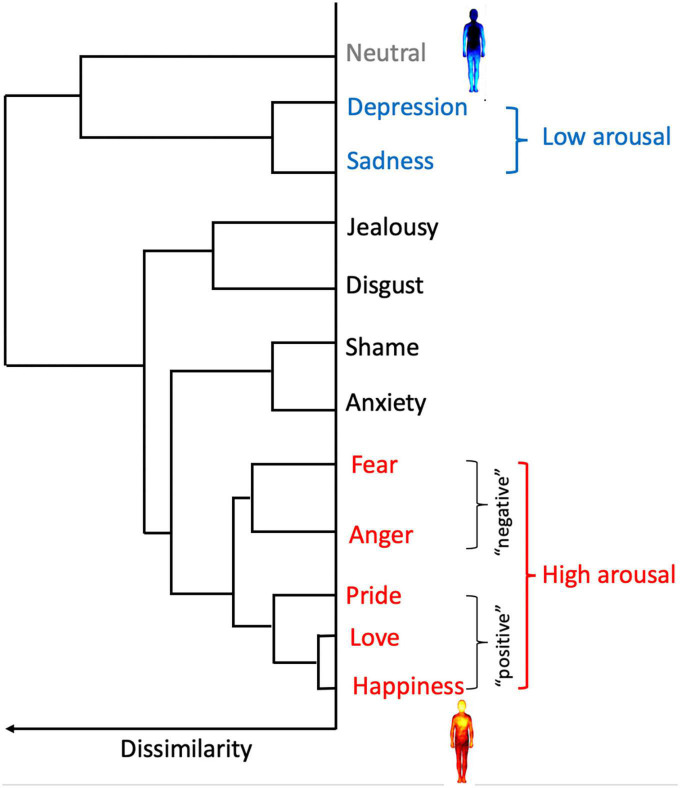
Hierarchical structure of the similarity between the bodily sensation of emotions. Sadness and depression formed a cluster that was clearly separated from all the other emotions (i.e., low arousal). Furthest from this low arousal cluster was a cluster of “positive” high arousal emotions (happiness, love, and pride) and a cluster of “negative” high arousal emotions (anger and fear). Low arousal emotions are experienced as bodily deactivation (i.e., blue in the map of depression) while high arousal emotions are embodied as bodily activation (i.e., red in the map of happiness). The two central clusters included anxiety, shame, disgust, and jealousy/envy. The neutral state was distinct from all other categories. This cluster analysis was used to form the low arousal (sadness and depression) and the high arousal (happiness, love, pride, anger, and fear) groups used in our study. Adapted from Nummenmaa et al. (2014).

**FIGURE 3:**
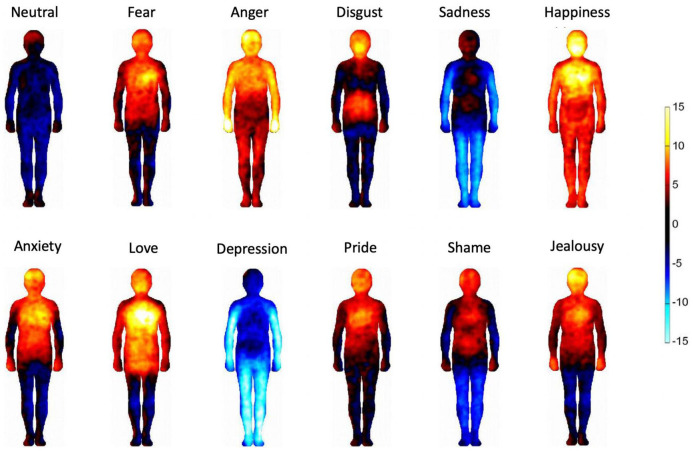
Bodily maps of emotions. The bodily regions whose activation was increased are depicted by warm colors and the bodily regions whose deactivation was increased are depicted by cool colors. The color bar indicates the *t*-statistic range.

In order to investigate the *congruency* of reported bodily sensations of emotions in relation to schizotypy, the strength (i.e., intensity) of activation and deactivation reported for low and high arousal emotions was assessed as a function of schizotypy. It is widely accepted that arousal/activation and valence are the two dimensions underlying the subjective experience of emotions (e.g., Lang et al., 1993; Posner et al., 2005; Colibazzi et al., 2010). By asking participants to report the activation and deactivation experienced in the context of different emotional experiences, the EmBODY task directly prompts for arousal (Hartman et al., 2021). We therefore used the hierarchical cluster analysis conducted by the original author group to categorize emotions into low and high arousal groups. Nummenmaa et al. (2014) found that the bodily sensations of emotions formed five clusters: at one extreme, lay a cluster of low arousal emotions (i.e., depression and sadness), while “positive” (i.e., happiness, love, and pride) and “negative” (i.e., anger and fear) high arousal emotions formed the two furthest clusters (see Figure 2). Notably, low arousal emotions are consistently experienced as bodily deactivation (i.e., blue), while high arousal emotions are reliably experienced as bodily activation (i.e., red) in previous body mapping experiments (Nummenmaa et al., 2014; Volynets et al., 2020) as well as in the current study (see Figure 3). Thus, in our study, depression and sadness formed the low arousal group, while the high arousal group consisted of happiness, love, pride, anger, and fear. The emotions that fell in the two middle clusters (i.e., shame, disgust, and jealousy), as well as the neutral state were discarded from this analysis.

In our analyses, activation reported in the context of a high arousal emotion (i.e., happiness, love, pride, anger, and fear) and deactivation reported in the context of a low arousal emotion (i.e., depression and sadness) are conceptualized as *congruent* bodily sensations of emotions, while activation reported in the context of a low arousal emotion and deactivation reported in the context of a high arousal emotions are examples of *incongruent* bodily sensations of emotions.

#### 2.2.2. Schizotypy

Schizotypy was assessed using the Schizotypal Personality Questionnaire (SPQ, Raine, 1991) and the Schizotypal Personality Questionnaire-Brief (SPQ-B; Raine and Benishay, 1995). The SPQ is a 74-item questionnaire designed to assess schizotypal traits based on DSM−III Schizotypal Personality Disorder criteria (American Psychiatric Association, 1980). The SPQ is the most widely used tool to assess schizotypy in research settings and has been shown to have excellent psychometric properties (e.g., Chapman et al., 1995; Wuthrich and Bates, 2005). It yields nine subscales: Ideas of Reference, Excessive Social Anxiety, Odd Beliefs/Magical Thinking, Unusual Perceptual Experiences, Odd/Eccentric Behavior, No Close Friends, Odd Speech, Constricted Affect, and Suspiciousness. A factor analysis revealed that these subscales best fit a three-factor model of schizotypy: Cognitive/Perceptual deficits, Interpersonal deficits, and Disorganization. The SPQ-B is an abbreviated version of the SPQ that yields scores for the each of the three factors of schizotypy (i.e., Cognitive/Perceptual, Interpersonal, and Disorganization). In our sample, 153 participants completed the SPQ and 266 completed the SPQ-B. In order to combine these two sub-samples, scores on the three factors of schizotypy were converted into *Z*-scores in each sub-sample such that each participant had a *Z*-score for each of the three schizotypy dimension, with higher scores indicating elevated schizotypy.

### 2.3. Data analysis

#### 2.3.1. Data preprocessing and visualization

Statistical analyses were implemented in MATLAB r2016a^1^ and R (Version 3.5.1, R Core Team, 2018). Bodily maps of emotions were generated for the whole sample using the method outlined by Nummenmaa et al. (2014). Briefly, the activation and deactivation reported for a given emotion were combined into a single map, and mass univariate *t*-tests were used to compare the activation/deactivation of each pixel against zero. A false discovery rate (FDR) correction with α = 0.05 was applied to account for multiple comparisons. *t*-values were used to generate maps where pixel coloration represents statistically significant bodily sensation in the context of an emotion. In these bodily maps of emotions, warm colors (i.e., red) represent bodily activation while cold colors (i.e., blue) represent bodily deactivation.

#### 2.3.2. Analysis of diffusion, size, intensity, clarity, and congruency metrics

##### 2.3.2.1. Diffusion

Given the nested nature of our dataset, linear mixed effect analyses with random intercepts for each participant, emotion, and ROI were used to assess the effect of schizotypy on the diffusion of the bodily maps using the *lme4* package (Bates et al., 2014). To estimate *p* values, we used a Satterthwaite approximation of degrees of freedom (Satterthwaite, 1941) and performed *t* tests with *lmerTest* (Kuznetsova et al., 2017). The absence of coloring within an ROI resulted in a diffusion score of 0 (25% of observations), which biased the dataset. To address this, a secondary analysis excluding zeros was conducted; the results remained unchanged.

##### 2.3.2.2. Size

Due to assumption violations for a linear regression model, the relationship between the size of embodied sensations of emotions and schizotypy was assessed using Spearman correlations, and a Holm correction was applied. The *DescTools* (Signorell, 2017) package was used to calculate Spearman correlations coefficients and their confidence intervals.

##### 2.3.2.3. Intensity

We first attempted to assess the link between schizotypy and the strength of emotional embodiment using a linear regression model, but diagnostic tests indicated that two assumptions (i.e., linearity and nature of the distribution of residuals) of the model were violated. Thus, the relationship between schizotypy and size of embodied emotions was assessed using Spearman correlations with a Holm correction.

##### 2.3.2.4. Clarity

To account for the count nature of the variable, its overdispersion, and excess zeros (i.e., most pixels were either not colored or colored only for activation or deactivation), the link between schizotypy and mixed pixels was assessed using a zero-inflated negative binomial regression in the *pscl* package (Zeileis et al., 2008; Jackman, 2020). Exponentiated coefficients were calculated to estimate odds ratios.

##### 2.3.2.5. Congruency

Violations of assumptions prevented the use of multivariate linear regressions to test whether schizotypy predicted the strength of congruent (i.e., activation for high arousal emotions and deactivation for low arousal emotions) and incongruent (i.e., deactivation in high arousal emotions and activation in low arousal emotions) bodily sensations of emotions. Instead, we used Spearman correlations with a Holm correction to assess the relationship between schizotypy dimensions and reported congruent/incongruent bodily sensation of emotions.

## 3. Results

Figure 3 shows the bodily maps of emotions of our sample. Emotions were associated with distinct bodily patterns of activation (red) and deactivation (blue) that are in accordance with previous cross-cultural results (Nummenmaa et al., 2014; Volynets et al., 2020). Emotions categorized as low arousal in subsequent analyses (i.e., sadness and depression) are experienced as bodily deactivation while high arousal emotions (i.e., happiness, love, pride, anger, and fear) are experienced as bodily activation. In contrast, the emotions that were excluded from the high/low arousal analysis (i.e., anxiety, shame, disgust, and jealousy) tend to be embodied a mix of activation and deactivation.

A mixed-effect analysis revealed no link between any of the three schizotypy factors and the diffusion of bodily sensations of emotions (Interpersonal: β = 0.02, 95% CI [−0.02, 0.07], SE = 0.03, *t* = 0.98, *p* = 0.33; Cognitive/Perceptual: β = 0.002, 95% CI [−0.05, 0.05], SE = 0.03, *t* = −0.07, *p* = 0.95; Disorganized: β = 0.005, 95% CI [−0.05, 0.04], SE = 0.03, *t* = −0.18, *p* = 0.86). Similarly, no link was found between any of the schizotypy dimensions and the proportion of the body colored (Interpersonal: *r*(417) = 0.02, 95% CI [−0.08, 0.11], *p* = 0.72; Cognitive/Perceptual: *r*(417) = −0.007, 95% CI [−0.10, 0.09], *p* = 0.88; Disorganized: *r*(417) = −0.02, 95% CI [−0.11, 0.08], *p* = 0.74). This remained true when grouping emotions into low (i.e., depression and sadness) and high (i.e., happiness, love, pride, anger, and fear) arousal. Thus, against our expectations, schizotypy was not associated with diffusion or size of embodied emotions.

A zero-inflated negative binomial regression revealed no effect of schizotypy on the presence (inflated model – Interpersonal: β = −0.24, 95% CI [−0.27, 0.30], SE = 0.16, *Z* = 1.57, *p* = 0.12; Cognitive/Perceptual: β = 0.02, 95% CI [−0.59, 0.07], SE = 0.15, *Z* = 0.14, *p* = 0.89; Disorganized: β = 0.13, 95% CI [−0.17, 0.43], SE = 0.15, *Z* = 0.91, *p* = 0.37) or number (count model – Interpersonal: β = 0.09, 95% CI [−0.34, 0.16], SE = 0.10, *Z* = 0.92, *p* = 0.36; Cognitive/Perceptual: β = −0.09, 95% CI [−0.16, 0.36], SE = 0.10, *Z* = 0.89, *p* = 0.37; Disorganized: β = 0.07, 95% CI [−0.27, 0.35], SE = 0.09, *Z* = 0.83, *p* = 0.40) of mixed pixels. However, when investigating high and low arousal emotions separately, we found a link between Interpersonal (i.e., negative syndrome) schizotypy and zero inflation (β = −0.28, SE = 0.12, 95% CI [−0.54, −0.03], *Z* = 2.25, *p*=0.02) for low arousal emotions such that negative schizotypy was associated with the presence of both activation and deactivation at the same location (i.e. mixed pixels). More specifically, the baseline odds of having no mixed pixels was 1.57 for low arousal emotions. This odd was decreased by 0.76 for each one-unit increase in negative schizotypy, suggesting that negative schizotypy was associated with the presence of mixed pixels. No link was found between schizotypy and the number (i.e., count) of mixed pixels in low-arousal emotions, or the presence or number of mixed pixels in high-arousal emotions.

A positive correlation was found between Interpersonal (i.e., negative) schizotypy and the intensity of bodily sensations of emotions [*r*(417) = 0.16, 95% CI [0.06, 0.25], *p* = 0.003]. Cognitive/Perceptual (i.e., positive) [*r*(417) = 0.07, 95% CI [−0.03, 0.16], *p* = 0.20] and Disorganized [*r*(417) = 0.08, 95% CI [−0.02, 0.17], *p* = 0.20] schizotypy did not significantly correlate with the intensity of painting. Similar results were found when investigating high and low arousal separately, such that only Interpersonal schizotypy predicted the intensity of both low [*r*(417) = 0.19, 95% CI [0.09, 0.28], *p* = 0.0002] and high [*r*(417) = 0.13, 95% CI [0.03, 0.22], *p* = 0.03] emotions.

Correlation analyses further revealed a link between schizotypy and incongruent bodily sensation of emotions (see Table 2). Specifically, we found that Interpersonal schizotypy correlated with both the amount of deactivation reported in the context of high arousal emotions [*r*(417) = 0.13, 95% CI [0.03, 0.22], *p* = 0.02] and the amount of activation reported in the context of low arousal emotions [*r*(417) = 0.12, 95% CI [0.02, 0.21], *p* = 0.05]. A link was also found between Cognitive/Perceptual schizotypy and the amount of deactivation reported in the context of high arousal emotions [*r*(417) = 0.11, 95% CI [0.02, 0.20], *p* = 0.05]. Interpersonal schizotypy was also linked to the amount of congruent bodily sensation reported for low arousal emotions [*r*(417) = 0.13, 95% CI [0.03, 0.22], *p* = 0.03].

**TABLE 2:** Correlations between schizotypy dimensions and congruent/incongruent bodily sensations of emotions.

	Congruent		Incongruent	
	**High arousal activation**	**Low arousal deactivation**	**High arousal deactivation**	**Low arousal activation**
SPQ interpersonal	0.10 [0.006, 0.19]	0.13* [0.03, 0.22]	0.13* [0.04, 0.23]	0.12* [0.02, 0.21]
SPQ cognitive/perceptual	0.03 [−0.06, 0.13]	0.02 [−0.07, 0.17]	0.11* [0.02, 0.20]	0.06 [−0.03, 0.16]
SPQ disorganized	0.06 [−0.03, 0.16]	0.07 [−0.03, 0.16]	0.06 [−0.03, 0.16]	0.05 [−0.05, 0.14]

Values reported in the table represent Spearman correlation coefficients and their 95% CIs. Asterisk indicate *p* < 0.05 after Holm correction.

## 4. Discussion

Schizotypy was not associated with the spread (i.e., diffusion), or size (i.e., proportion of the body colored) of embodied sensations of emotions. However, negative schizotypy correlated with an increased intensity of bodily sensations of emotions, and this remained true when exploring high and low-arousal emotions separately. This finding goes against the conventional expectation that negative syndrome is associated with decreased emotional experiences and expression. In fact, our results suggest that negative syndrome is associated with more intense experiences of emotions at the bodily level, although the expression of such emotions may not be readily captured by conventional methods. Negative schizotypy also predicted the presence of mixed pixels for low-arousal emotions. In line with our hypothesis, we found that schizotypy predicted incongruent bodily sensations of emotions (Table 2). Specifically, negative schizotypy predicted bodily deactivation felt in the context of high-arousal emotions and bodily activation during low-arousal emotions. Positive schizotypy also predicted deactivation during high-arousal emotions. In sum, individuals with higher negative schizotypy reported experiencing embodied emotions with higher intensity and lowed clarity (i.e., more mixed pixels), and endorsed more incongruent bodily sensations of emotions (the latter was also true for those with high negative schizotypy). As expected, some of these differences were particularly notable for low-arousal emotions.

The nature or impact of the emotional embodiment differences related to schizotypy remains to be investigated. In fact, given the results of a recent study documenting no link between schizotypy and interoceptive functioning (Torregrossa et al., 2022), it seems unlikely that the increased intensity of bodily sensations reported by individuals with elevated negative schizotypy results from a heightened awareness or attention to bodily signals in this population. We also contrast this finding with that of a study that found decreased intensity of embodied emotions in individuals with alexithymia (Lloyd et al., 2021). It is therefore possible that the increased intensity of embodied emotions experienced by individuals with high negative schizotypy facilitates emotion identification. In line with this hypothesis, a recent study documented a potential protective effect of negative schizotypy on disturbances of body ownership (Torregrossa and Park, 2022). However, our results also indicated that individuals with elevated schizotypy experience more incongruent (e.g., activation in the context of a low arousal emotion, or deactivation in the context if a high arousal emotion) and blurred (i.e., activation and deactivation being experienced simultaneously in a given bodily region) bodily sensations of emotions, which could in turn lead to incorrect emotion identification. Therefore, the functional impact of emotional embodiment differences along the schizotypy spectrum remains to be investigated. Specifically, future research assessing emotional embodiment in conjunction with other aspects of emotional and social functioning (e.g., alexithymia, emotional awareness, and emotion identification) is needed to shed light on the functional implication of our findings.

In comparing the present results to those of a previous study investigating emotional embodiment in people with schizophrenia (Torregrossa et al., 2019b), we note key parallels and differences. Torregrossa et al. (2019b) found that individuals with schizophrenia reported more deactivation in the context of high-arousal emotions; the present study revealed the same tendency in individuals high schizotypy. Similarly, in line with the higher number of mixed pixels recorded in individuals with schizophrenia (Torregrossa et al., 2019b), the present study documented a link between negative schizotypy and the presence of mixed pixels for low arousal emotions. We also note that the emotional embodiment differences observed along the schizotypy spectrum were particularly pronounced for low-arousal emotions, which echoes the specific alteration of embodiment for low-arousal emotions in schizophrenia (Torregrossa et al., 2019b). On the other hand, Torregrossa et al. (2019b) found no difference in the intensity (Intensity is referred to as “magnitude” in Torregrossa et al., 2019b) of embodied emotions reported by individuals with schizophrenia and control participants, in contrast to the present study, which found a link between negative schizotypy and the intensity of bodily sensations of emotions. Additionally, though Torregrossa et al. (2019b) did not compute the diffusion or size (i.e., proportion of the body colored) of the embodied emotions in their sample, their topographical maps suggested that individuals with schizophrenia endorsed bodily sensations of emotions that were more spread out than controls’. In the present study, we found no link between schizotypy and the diffusion or size of emotional embodiment. In addition to quantitative and/or qualitative differences between schizophrenia symptomatology and schizotypy traits, the very large sample size for the current study might explain some of these differences.

These discrepancies make it difficult to assess whether the differences in emotional embodiment found in individuals with elevated negative schizotypy represent the emergence of anomalous bodily sensations of emotions documented in schizophrenia (Torregrossa et al., 2019b). In fact, most individuals with high schizotypy will not go on to develop a psychotic illness (e.g., Debbané et al., 2015). Investigation of embodied emotions in individuals at clinical high risk for a psychosis would help clarify whether the differences in emotional embodiment associated with negative schizotypy reported here represent early disruptions of the bodily self or clinically insignificant individual differences. In addition, a longitudinal design will be needed to determine whether differences in emotional embodiment in young adults with elevated schizotypy represent a prodromal sign for risk for psychosis. Studies investigating emotional embodiment across the psychotic spectrum (i.e., in prodromal individuals and people experiencing the first episode of psychosis) are also needed to clarify the developmental trajectory of anomalous emotional embodiment in the schizophrenia spectrum.

The clinical implications of our findings extend beyond the schizophrenia spectrum. In fact, as previously detailed, disruptions of emotional embodiment likely impact one’s ability to verbalize or label emotions (i.e., alexithymia), which is common in a wide spectrum of conditions including affective disorders and schizophrenia. These disruptions in emotional functioning present a major barrier to successful engagement in psychosocial treatment, forming and maintaining social relationships and being able to have insight into one’s conditions. Thus, improving one’s awareness of the bodily sensations accompanying their emotions could help toward therapy outcomes and successful social relationships across a variety of conditions. Although, to our knowledge, no intervention directly targeting emotional embodiment exist at present, mindfulness-based and body-centered interventions might be good candidates. In fact, a recent meta-analysis showed a small but significant effect linking mindfulness to body awareness (Treves et al., 2019). A recent review also revealed a robust effect of a variety of body-centered interventions on both physical and psychological symptoms across a variety of clinical populations (Tarsha et al., 2020).

Despite the large sample size, and novel and noteworthy results of our study, there are limitations. First, differences in methodology prevent important comparisons between our work and previous body mapping studies that examined individuals diagnosed with schizophrenia. For instance, as previously mentioned, Torregrossa et al. (2019b) did not compute the proportion of the body colored or diffusion of the bodily sensations of emotions. However, Torregrossa et al. (2019b) used a linear discriminant analysis (LDA) to test whether different emotions were associated with statistically different bodily sensations in schizophrenia and found a reduced classification accuracy in schizophrenia compared to control individuals. The dimensional nature of our dataset prevented the use of LDA to assess the independence of embodied emotion in relation to schizotypy. Additionally, given the nature of our sample (i.e., undergraduates at a highly selective university), it is possible that the distribution of schizotypy scores is skewed such that the higher end of spectrum is under-represented, and it is possible that the qualitative experience of schizotypy would be different in a general population or a clinical sample reporting similar levels of schizotypy. Our decision to investigate the schizotypy spectrum dimensionally (i.e., general population) also prevents us from drawing definitive conclusions about the link between differences in emotional embodiment in people with high schizotypy and in individuals with diagnosed with schizophrenia (Torregrossa et al., 2019b). In addition, it is possible that demographic factors (e.g., gender and race/ethnicity) impact the relationship between schizotypy and embodied emotions. In fact, we found gender and race/ethnicity differences in levels of schizotypy and, although preliminary *post hoc* analyses did not indicate an effect of demographic variables on the results reported in this study, future work directly aimed at investigating the impact of demographic factors on emotional embodiment is needed to rule out this possibility. Lastly, we did not measure alexithymia or other aspects of emotional or social functioning, which limits the interpretations of our results regarding the functional impact of the observed differences in embodiment emotions along the schizotypy spectrum.

## 5. Conclusion

In sum, this study examined, for the first time, emotional embodiment in relation to schizotypy. We found that individuals with elevated negative schizotypy experience the bodily sensation of their emotions with greater intensity but reduced clarity (for low arousal emotions). We also found that both positive and negative schizotypy predicted incongruent bodily sensations of emotions (e.g., bodily activation felt in the context of a low arousal emotion). Importantly, some aspects of emotional embodiment (i.e., the diffusion and size of bodily sensations) did not systematically vary as a function of schizotypy. These novel results add to the recent body of literature documenting both intact (e.g., interoception; Torregrossa et al., 2022) and mildly disrupted (e.g., body ownership; Torregrossa and Park, 2022) aspects of the bodily self experience in relation to multidimensional schizotypy. More work is needed to understand the implications of these results in terms of emotional and social functioning as well as the possible role of emotional embodiment in psychosis-risk.

## Data availability statement

The raw data supporting the conclusions of this article will be made available upon request by the authors, without undue reservation.

## Ethics statement

The studies involving human participants were reviewed and approved by the Vanderbilt University Institutional Review Board. The patients/participants provided their written informed consent to participate in this study.

## Author contributions

SP and LT contributed to conception and design of the study. LT, MS, and SB collected the data, organized the database, and performed the statistical analysis. MS developed a method to extract a parameter. LT wrote the first draft of the manuscript. LT, SP, SB, and MS wrote sections of the manuscript and worked on subsequent drafts. All authors contributed to manuscript revision, read, and approved the submitted version.

## Footnotes

1
www.mathworks.com
